# Active lead-in variability affects motor memory formation and slows motor learning

**DOI:** 10.1038/s41598-017-05697-z

**Published:** 2017-08-10

**Authors:** Ian S. Howard, Christopher Ford, Angelo Cangelosi, David W. Franklin

**Affiliations:** 1 Centre for Robotics and Neural Systems, School of Computing, Electronics and Mathematics, University of Plymouth, Plymouth, United Kingdom; 2 Neuromuscular Diagnostics, Department of Sport and Health Sciences, Technical University of Munich, Munich, Germany

## Abstract

Rapid learning can be critical to ensure elite performance in a changing world or to recover basic movement after neural injuries. Recently it was shown that the variability of follow-through movements affects the rate of motor memory formation. Here we investigate if lead-in movement has a similar effect on learning rate. We hypothesized that both modality and variability of lead-in movement would play critical roles, with simulations suggesting that only changes in active lead-in variability would exhibit slower learning. We tested this experimentally using a two-movement paradigm, with either visual or active initial lead-in movements preceeding a second movement performed in a force field. As predicted, increasing active lead-in variability reduced the rate of motor adaptation, whereas changes in visual lead-in variability had little effect. This demonstrates that distinct neural tuning activity is induced by different lead-in modalities, subsequently influencing the access to, and switching between, distinct motor memories.

## Introduction

We continuously account for variations in the dynamics of our environment and bodies in order to perform tasks effectively. The rate at which we can compensate for such environmental changes and learn new skills plays an important role in our performance. Consequently, factors that affect learning rate are of interest in sports training as well as movement rehabilitation. Many studies have utilized novel viscous force fields implemented on robotic devices in order to examine the factors that affect the learning and recall of motor memories^1, 2^. When two opposing force fields are randomly switched, interference arises preventing any learning^1, 3–5^ unless additional contextual information is available^6–12^. As only specific types of contextual information allow the learning of two opposing force fields^11^, this suggests that only specific signals are able to switch between or access specific motor memories in a given task. Since natural movements are often concatenations of several sub-movements, we previously extended the examination of viscous force field adaptation to two-part movements. Using interference studies we showed that strong contextual effects arise from distinct lead-in movements occurring immediately prior to the adaptive movement can strongly affect memory learning and recall^13^. Interestingly past contextual movement is equally effective at reducing the interference irrespective of whether it is actively generated, passively performed, or visually observed^13^. Further investigations have subsequently showed that each of these types of contextual lead-in movements exhibit different degrees of angular generalization, with active and visual lead-in movements showing deep and narrow or shallow and wide tuning respectively^13–15^.

Contextual effects that influence motor memory are not limited to lead-in movements, as distinct follow-through movements also exhibit similar effects^16, 17^. More interestingly, changes in the diversity of follow-through affected the learning rate of a single dynamic force field^16^. In particular, a single consistent follow-through resulted in faster learning than variable follow-throughs. We proposed that this phenomenon arose because the follow-through movement affects the specific motor memory that is activated for the learning and recall. That is, movements with a consistent follow-through are stored within a single motor memory. In contrast, in the case of variable follow-throughs, the learning is stored in multiple motor memories, thereby reducing the rate of learning. Here we examine whether variability in lead-in movements also modulates the learning rate in a similar fashion. We propose that if our explanation of the mechanism behind the difference in learning rate is correct, then the learning rate will be strongly dependent on the angular generalization that contextual movement produces. That is, variability would have little affect on the learning rate if contextual movements exhibit a broad tuning, but it will do so if their turning is narrow. As visual and active lead-in movements show different generalization of tuning^13–15^, we therefore predict that these two types of contextual movements would produce different learning rates. To test this hypothesis, we examine how the variability of lead-in movements across active and visual lead-in modalities affects the dynamic learning rate in the subsequent movement.

## Results

It has been shown that the variability of follow-through movements affects the rate of motor memory formation and the specific motor memory in which this learning is stored^16^. Past movement similarly influences motor memory formation and recall in subsequent movements, behaving like a contextual cue, provided that the movements are closely linked in time^13^. However different lead-in movement modalities exhibit different properties. For example, the angular generalization of active lead-in movements is narrower and deeper than that of visual lead-in movements^15, 18^. We formulate a single rate state-space model to predict how contextual movement tuning widths and lead-in movement diversity should affect the learning of a novel dynamic force field. State-space models have been proposed previously to explain the behavior seen in dynamic learning tasks^19, 20^. Contextual learning effects can be represented in these models by using an explicit state for each context and appropriately weighting its contributions to the learning and recall process as a function of trial context^21, 22^. When contextual effects are discrete and non-overlapping, binary weighting factors can capture these contextual dependencies. However, in our case contextual effects arise due to different lead-in movement directions. In order to model the angular dependency we use a set of basis functions, each of which has an angular receptive field that overlaps those from neighboring receptive fields. To capture the circular nature of the lead-in movements, we model the angular tuning characteristic of each base using a von Mises function with appropriate tuning width, previously adopted to model visual contextual tuning responses^18^.

In the formulation of our state-space model, there are characteristics from the behavioral data of prior work that need to be captured^16^. In that study, as the angular diversity of contextual movements increased, the overall learning rate for a specific lead-in direction decreased. In addition, although changes in diversity resulted in changes in learning rate, the final asymptotic level of compensation remained independent of this lead-in diversity. The implications of these observations are twofold. First, diverse lead-ins should result in a spread of activation across more states, thereby requiring more memories to learn. Second, on any given lead-in trial, the memory of states that are not activated (the lead-ins do not fall within their receptive fields) should not decay. This requires that the retention term in the state space module formulation is a function of contextual activity, as proposed previously for object manipulation^23^.

**Figure 1:**
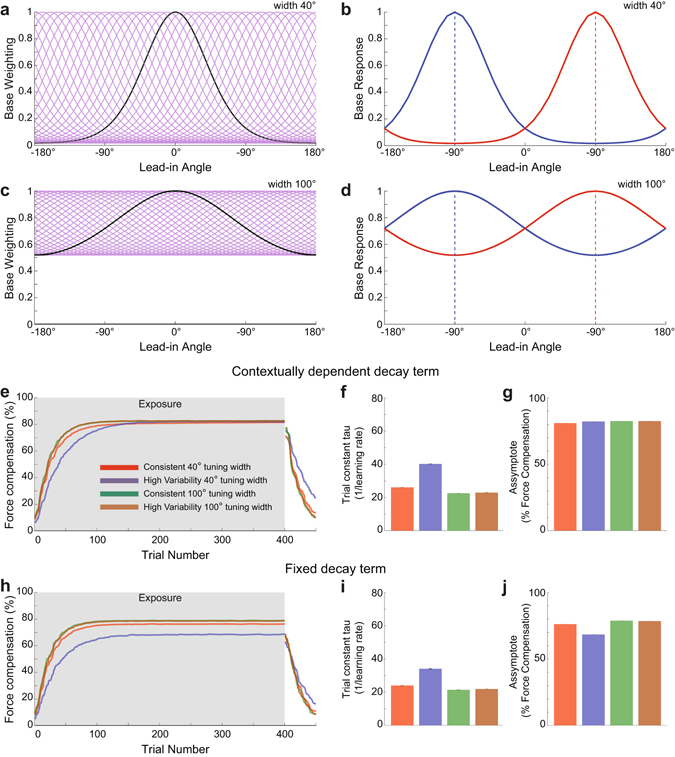
Models of different neural tuning widths predict different speeds of motor learning for movements with a range of lead-in movements. (**a**) Receptive fields and their responses to contextual lead-in movements were modeled with von Mises basis functions. Thirty-six narrow basis functions (σ = 40°) uniformly spanning 360° range were used to model active lead-in movements. The response of a single basis function centered at 180° is highlighted in black to illustrate the shape of each function. (**b**) Basis function activation response to contextual lead-in movements at 90° and 270° plotted in blue and red retrospectively. The two movements almost activate a different set of basis functions. (**c** and **d**) Wide von Mises basis functions (σ = 100°) to model visual lead-in movements. The basis functions activated by the two movement directions overlap. (**e**) Simulation of the effect of consistent and diverse lead-in movements with narrow and wide receptive fields using a context-dependent retention term. Red shows low diversity lead-in with narrow 40° tuning, blue shows high diversity lead-in with narrow 40° tuning, green shows low diversity lead-in with wide 100° tuning and brown shows high diversity lead-in with wide 100° tuning. (**f**) The mean and SE for exponential fit parameters trial constant tau. (**g**) The mean and SE for exponential fit parameters asymptote coefficient. (**h**–**j**) Simulation of the effect of consistent and diverse lead-in movements and corresponding bar graph of parameters as in (**e**–**g**), but using a fixed (non-contextually dependent) retention term.

To illustrate the model, we consider a scenario in which there are two lead-in directions, and examine the consequences of using narrow (σ = 40°) or wide (σ = 100°) basis functions (Fig. 1a and c). The spread of training trials across the two different lead-in directions (90° and 270°) results in two separate basis function activation patterns, the peak location of which depends on the lead-in angle (Fig. 1b and d). Each response is determined by the amount the given lead-in movement falls into a given base’s receptive field. It can be seen that the overlap increases as the basis function widens. Therefore, a narrow tuning leads to more distinct state activation for each of the two lead-ins and consequently less sharing of basis functions than wide tunings. Thus, learning a single force field with narrow tuning with two widely separated lead-in movements becomes similar to performing two separate motor learning tasks rather than a single task, since each of the movement directions will activate separate motor states. As there are more separate states to learn, and each can only be learned on trials in which they are activated, training will take a larger number of trials, reducing the overall learning rate. In the presence of narrow receptive fields, increasing the spread of lead-in movements will similarly play a role in reducing learning rate; a single lead-in will result in only a single activation pattern whereas multiple lead-ins may lead to multiple distinct activation patterns.

We tested the model on simulated data representing field exposure for single and diverse lead-in movements with narrow and wide contextual tuning characteristics. To do so we generated 450 trials of data with a pseudo-randomly located simulated channel trial every 10 trials. We generated 12 sets of data to represent 12 runs with virtual participants for each of four experimental conditions representing narrow and wide receptive fields with either consistent or diverse lead-in movement angles. The first 400 trials were force field trials and the last 50 trials were null trials to simulate the final washout condition. On the basis of previous studies, we selected either a single lead-in movement at 135° or one-of-nine lead-in movements over a 180° range. The diverse lead-in condition used a 180° range of starting locations (rather than 360°) since this provided lead-in movements with wide variations, but without obscuring the final target location with the start location. The consistent lead-in condition used a single off-center movement at 135° to avoid a direct straight-through movement to the target and thus make it more comparable with movements made in the diverse variability condition. To represent the tuning characteristics of the receptive fields, we used von Mises functions with no offset, scaled to unity at their peaks with the conservative estimates for active and visual contextual tuning with equivalent standard deviation values of σ = 40° and σ = 100° respectively. Learning rate and retention parameters were estimated by fitting a single rate model to the results of control Experiment 3, which consists of a single dynamic learning task without any lead-in movement. The estimated parameters are a retention rate of 0.99 and a learning rate of 0.052, which were used for all simulations.

For all simulated conditions the mean and SE was computed across 12 runs (to simulate 12 participants) in the same fashion as for experimental data. In the narrow tuning lead-in condition, high variability of lead-in movements shows slower learning than achieved using a single consistent lead-in (Fig. 1e–g). However in the wide tuning lead-in condition, changes of lead-in movement variability or diversity have little effect. Based on these simulations we predict that diversity in active lead-in movements would produce a larger effect of the learning rate than diversity in visual lead-in movements.

In order to demonstrate the necessity of using a retention term in the state space model that is contextually dependent, we also simulated the same design but with fixed retention rates with no contextual dependence (Fig. 1h–j). The corresponding predictions demonstrate that the model no longer predicts the same asymptotic levels of compensation for different lead-in diversities.

**Figure 2:**
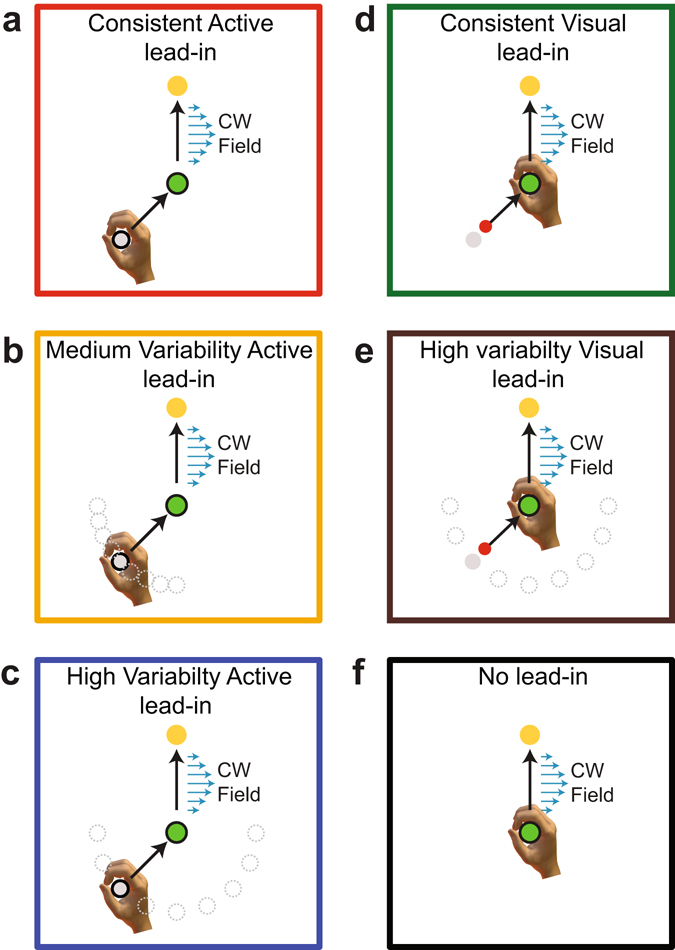
Experimental paradigm in which the effects of sensorimotor modality and lead-in variability on curl field adaptation learning rate were examined. Participants performed a two-part movement, with an initial lead-in motion to the central target (green) followed by an adaptation movement on which a curl force field (blue arrows) was applied. The direction of curl force field and lead-in movements were counterbalanced across participants. In all three experiments channel trials were used on the adaptation movement to assess learning rate. (**a**) Consistent active lead-in condition. The participant makes an active movement from the starting location (grey circle) to the central location (green circle). This is immediately followed by an active movement to the final target location (yellow circle). A single starting location was used, located at either 45° or −45° (counterbalanced across subjects). (**b**) In the medium variability active lead-in condition, the starting location was pseudo-randomly selected from 1 of 9 locations spanning the range of 0° to 90° (or 0° to −90° for half of the subjects). (**c**) In the high variability active lead-in condition, the starting location was pseudo-randomly selected from 1 of 9 locations spanning the range of −90° to 90°. (**d**) Consistent visual lead-in condition. This condition is similar to the active consistent condition, with the exception that the lead-in motion consists only of a visual motion of the cursor from the starting location (grey shaded circle) to the central location while the hand remains stationary at the central location. Only a single starting location was used (45° or −45° counterbalanced across subjects). (**e**) In the high variability visual lead-in condition, the starting location was pseudo-randomly selected from 1 of 9 locations spanning the range of −90° to 90°. (**f**) In the control condition, there was no prior lead-in movement.

**Figure 3:**
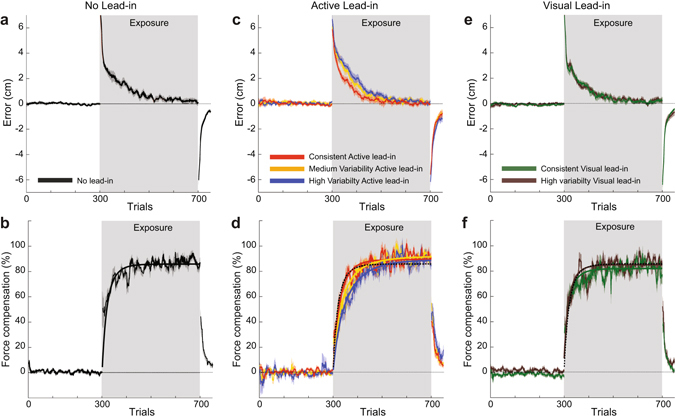
Variability in the direction of active lead-in movements slows learning rate. (**a**) The kinematic error (ten-trial running mean ± SE across participants) for the no lead-in condition. The grey background shading indicates the exposure phase. (**b**) The percentage of force compensation for the no-lead-in group. The solid line is the mean of the best-fit exponentials to individual participant data. (**c**) The kinematic error for the consistent active lead-in (red), the medium variability active lead-in (orange) and high variability lead-in (blue) groups of subjects. (**d**) The force compensation for the active lead-in groups. (**e**) The kinematic error for the consistent (green) and high variability (brown) visual lead-in groups. (**f**) The force compensation for the visual lead-in groups.

In order to test this experimentally, participants adapted to a curl force field while performing two-part movements comprised of a lead-in movement followed by an adaptation movement on which the force field was applied (Fig. 2). The lead-in movement could be either active (3 groups) or visual (2 groups), and was performed either from a consistent or a variable starting location. A control group was also examined which had no lead-in movement. This provided a total of six different experimental conditions. In all groups, prior to field exposure, the kinematic error remained low (Fig. 3a,c,e) and the force compensation was close to zero (Fig. 3b,d,f). A comparison in the kinematics of the adaptation movement was performed for the second half of the pre-exposure phase (trials 150–300) across all experimental groups. There were no differences in the dwell time (F_4,55_ = 0.271; p = 0.89), or lateral deviation (F_5,66_ = 1.445; p = 0.22) across conditions. However, there was a significant effect of peak speed (F_5,66_ = 7.514; p < 0.001). Post-hoc comparisons showed that there were no significant differences between any the active groups (all p > 0.9), and no significant differences between any of the visual and control groups (all p > 0.9). However there were differences between several of the conditions with all visual and control groups moving significantly faster than either the consistent or highly variable active groups (p < 0.05). Differences in the speeds between all groups were on the order of 10% (ranging between 70.8 and 78.6 cm/s). Differences in the speeds within each sensory modality were lower, ranging between 70.8 and 73.4 cm/s for the active condition and 77.5 and 78.6 cm/s for the visual condition. As the measure of force compensation, calculated by regressing the measured force against the velocity, is insensitive to variations in movement speed, these differences are expected to have little effect on the overall experimental results, and even less on the critical comparisons of the effect of lead-in diversity within a given modality. This is because it is the inter-modality comparisons which are the quantities of most interest, since these demonstrate the effect of changes in diversity on learning rate.

In all groups, at the onset of field exposure the kinematic error was increased dramatically, and then gradually reduced during repeated exposure. This reduction in error was paralleled by a steady increase in the level of force compensation, however the exact evolution of these measures varied with the lead-in conditions. At the onset of the post-exposure phase, all groups exhibited large kinematic errors in the opposite direction to the initial exposure phase, which reduced rapidly over subsequent trials. This was associated with a similar reduction in force compensation. The control condition with no lead-in movement shows all of these expected characteristics of the learning process (Fig. 3a,b). The best-fit exponential function to the force compensation data is shown with the solid line (Fig. 3b).

The first experiment examined the effect of active movement lead-in diversity (Fig. 3c,d). The lead-in movements were all active, but varied in terms of the variability, being either consistent (Fig. 2a), of medium variability (Fig. 2b) or high variability (Fig. 2c). During the exposure phase, the kinematic error in the consistent active lead-in condition (red) reduces quicker than the medium variability active condition (orange), which in turn reduces quicker than the high variability condition (blue). A similar effect was found in the rate of rise of force compensation for all three groups (Fig. 3d). The fastest increase in force compensation occurs in the consistent condition (red), then the medium variability condition (orange), and finally the high variability condition (blue). Moreover, it can be seen that the washout behavior in the post-exposure phase is qualitatively similar to both the rate of adaptation and the model predictions, with slower de-adaptation seen for the high variability condition (Fig. 3d).

In the second experiment a corresponding examination of the effect of visual movement lead-in diversity was examined, with two levels: consistent and high variability. Both groups of subjects had similar rates of changes in kinematic error (Fig. 3e) and force compensation (Fig. 3f) during the exposure phase.

**Figure 4:**
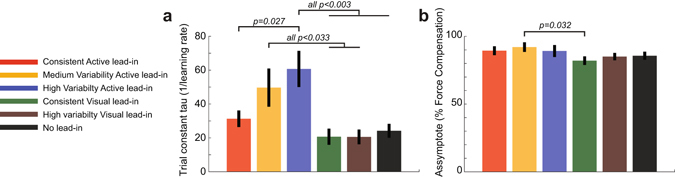
Comparison of learning parameters across the experimental conditions. (**a**) The trial constant tau for all six conditions in the three experiments. Values are the mean and SE across subjects for the exponential fit parameters to the force compensation during the exposure phase. All statistical differences between conditions based on post-hoc comparisons are shown. (**b**) The asymptote coefficient for the exponential fit parameters to the force compensation data.

Across all three experiments (comprising 3 active, 2 visual and 1 control groups, providing six experimental conditions in total) there were no significant differences in the final level of kinematic error across the last 20 exposure trials (F_5,66_ = 0.28; p = 0.93) or level of force compensation across the last 10 channel trials (F_5,66_ = 2.203; p = 0.064) in the exposure phase. In order to examine the effects of both the sensory modality and diversity of the lead-in movements on learning rate, the exponential parameters were compared across all six conditions in the three experiments (Fig. 4). The trial constant tau (inverse of learning rate) was significantly different across the conditions (F_5,66_ = 6.541; p < 0.001). Post-hoc comparisons showed that there were no significant differences between any of the two visual conditions, the consistent active condition, or the control condition (all p > 0.36). However, the medium variability active lead-in condition was significantly slower than the two visual conditions (p < 0.033) and the high variability active condition was significantly slower than the consistent active condition, both visual conditions, and the control condition (all p < 0.028). Therefore, only in the active lead-in condition did lead-in variability slow the learning rate. An ANOVA on the final asymptote level of force compensation found a significant main effect (F_5,66_ = 2.506; p = 0.039). However post-hoc tests indicated that the only significant effect was that consistent visual condition was slightly lower than the medium variability active lead-in conditions (p = 0.032). All other conditions were not significantly different from each other (all p > 0.2).

Overall the results show that the high variability active lead-in exhibits slower learning than that of a consistent lead-in or the control condition. However no such effect for the lead-in variability is seen for the visual lead-in movements. These experimental results are consistent with the model predictions. Namely that active contextual movements, with their narrow generalization characteristics^15^, will show a reduction in learning rate as the diversity of the lead-in movements increases. In contrast, visual lead-in movements, with their wider generalization characteristics^18^, will show little difference in learning rate.

## Discussion

In this study we examined how changing the diversity (variability) of lead-in movements affected the rate of dynamic learning in an immediately following adaptation movement. Using a curl field learning paradigm, we investigated if consistent lead-in movements provided faster learning than diverse lead-in movements using a task in which starting points varied from trial to trial. The effect of diversity was examined for both active and visual lead-in sensorimotor modalities. Changing the lead-in movement diversity produced significant differences in the learning rate when the lead-in was actively performed, but not when visual in nature. There was also no difference between any of the consistent lead-in movements and the no lead-in control condition, indicating that although variable lead-ins can slow learning, consistent lead-ins do not speed-up learning compared to a no lead-in condition. These experimental results were consistent with computational predictions based on differences in the generalization characteristics that arise from active and visual lead-in movements. This shows that both sensorimotor modality and diversity of lead-in movement have a strong effect on rate of acquisition of a motor memory.

Generalization is an important characteristic of motor learning. Generalization involves making use of the learning obtained in a specific context or movement, to a range of different movements or other contexts. That is, when we adapt to novel dynamics at a given location in space relative to our body and in a given direction, some of this learning - but not all of it - transfers to other movement locations and other movement directions^24–27^. Although adaptive motor memories generalize to new movements, the effect decreases as the movement deviates further from the originally learned movement. Such observations suggest that the learning was performed with a set of neural basis functions or motor primitives in velocity space^19, 20^. Within this framework, when motions are close to the originally learned movement, the sensorimotor system weights the associated basis functions by distance in state space, using the previously modified neural basis functions, and activating part of the learned motor memory. In our task, generalization is exhibited about the lead-in movement direction^14^, resulting in higher levels of predictive force compensation when the lead-in movement is close to the direction at which the task was originally learned. It is possible to interpret these results as specific lead-in movements activating local basis functions that are associated with the learned motor memory. Alternatively, recent studies have suggested that the motor system should be thought of as a dynamical system in which the initial neural activity sets up the movements to evolve through the unveiling of the neural dynamics^28–30^. Different initial neural states produced through different initial lead-in movements would therefore produce a different set of neural dynamics, in which opposing dynamics could be learned^17^. We can therefore interpret our study as different lead-in movements producing different set of neural dynamics, each of which learns the same dynamics, and thereby slowing the learning of the novel dynamics.

It has been shown that different lead-in modalities exhibit different contextual generalization functions^15, 16, 18^. After learning a single force field, changes in the active lead-in direction affect the memory recall of learned dynamics more than corresponding changes in visual motion^15, 18^. Thus the predictive force drops as the lead-in movement direction is moved further from the learned direction, but less so for visual motion compared to active movements. In addition, these two types of contextual lead-in movements exhibit different modulations depths. We hypothesized that as a consequence of these different characteristics, varying active lead-in movement direction would result in a stronger modulatory effect on motor learning and recall than would varying visual lead-in movement direction. Therefore when active lead-in movements are diverse and sufficiently spread out, each lead-in motion will effectively partition the memory used to represent the compensation learned in the subsequent movement, resulting in slower learning. We found that this was indeed true for the active lead-in movements, with the large variability lead-in condition showing slower learning than the consistent or no lead-in movement conditions. However, this difference in the learning rate was not seen in the visual conditions, as predicted by the generalization characteristics of the visual lead-in movements.

In order to predict how lead-in variability and tuning characteristics might affect learning rate, we made use of a single-rate state space model. To capture the contextual effect of lead-in direction, we used a von Mises tuning function, allowing us to model the generalization properties of the motor memories as the lead-in motion changes. Our simulations were made using previous conservative estimates of σ = 40° and σ = 100° for active and visual lead-in generalization tuning^15, 18^. Based on these parameters, the model predicted an effect of lead-in diversity for active movements, but not for visual lead-in movements. We note that more extreme estimates for the active and visual tuning width obtained in some prior experimental conditions, namely of σ = 30° and σ = 600°, lead to an even wider divergence between the consistent and variable lead-in conditions. Although this model of adaptation, built upon the basis of previous experimental results^14, 15, 18^, is consistent with the experimental results, it is always possible to come up with alternative models that would make similar predictions. The simplicity of our proposed model is that all of the necessary parameters can be obtained directly from previously published results.

In our state space model, a mixture of von Mises basis functions are tiled across the state space. This approach of building-up a complex overall response using a combination of simple processing elements, has its roots in the mixture of experts model^31, 32^. The mixture of experts was originally proposed to tackle supervised learning tasks, making use of a set of basis functions coupled with a non-linear gating mechanism, used during both learning and recall in order to divide up a complex task into simpler subtasks. This ensures that only a fraction of the experts are active in a given context, such that different subsets of experts can be locally specialized to fit smaller regions of a complex dataset. By turning off irrelevant basis functions during learning, gating ensures memories associated with them are protected.

A similar approach to the mixture of experts, applied to human motor control, is the MOSAIC model^33, 34^. This makes use of multiple paired forward–inverse models. Within the MOSAIC framework, a responsibility signal is estimated on the basis of sensory input cues as well as from the prediction error arising from actual state and the state estimated by the forward models. The responsibility signal is used to gate the output of the inverse model used in the control task as well as guiding the learning process of the paired forward–inverse models. As with the mixture of expert’s model, the gating signal ensures only the appropriate modules are updated and that memories in task-irrelevant modules are protected and remain unaffected.

In our model, the context (angle of lead-in movement) is not only used to modulate the rate of learning, but also to gate the retention in order to protect inactive memories. The retention rate, therefore, approaches unity for irrelevant contexts such that their memories are retained without decay.

Although motor memory learning and formation is widely accepted as being a context dependent process, there is some dispute in the literature regarding the role played by context in memory decay. As pointed out^23^, although many state space models developed to model motor learning use contextually dependent learning rates, they have assumed context independent retention^9, 19–22, 35–37^. This implies that any memory not currently activated, decays whenever another motor memory is used, regardless of its similarity to the activated motor memory. For example, the model proposed by Lee and Schweighofer incorporates a context-independent fast process and multiple context-dependent slow processes^22^. Contextual cue input selects the appropriate states to be summed in the total output and allows the motor errors to update the activated states from motor errors. Consequently any inactive memory is not protected against decay, since the forgetting factor for the inactive states is not contextually gated and therefore the corresponding values will gradually be forgotten over time.

However, it has been shown that object manipulation and dynamic learning movement tasks are both consistent with context dependent decay^23^. Ingram and colleagues suggest that a context dependency of retention may provide a means to optimize motor control since it can provide a means to reduce unnecessary force output when it is not required, but still protects newly forming memories which are not currently activated. This view is also supported by a computational study which proposed that context-dependent memory decay is the result of effort minimization^38^.

In our simulations, there was a clear requirement for a context-dependent retention term in our state space model. More specifically, it was necessary to make the retention term contextually dependent in order to account for the observation seen in both previous^16^ and current results, that the asymptotic level of compensation was independent of follow-though variability, and independent of lead-in variability in the current study. This suggests that the motor memory from a given lead-in movement direction is retained while a different lead-in movement activates a different motor memory.

The predictive output of motor memories is normally considered to decay towards baseline in the absence of error^39^, in order to minimize energy expenditure^40, 41^. Recently it has been proposed that decay of a particular motor memory only takes place when the context of movement changes^42^. In their study, introducing noisy channel trials that mimicked the natural variability of movements stopped the decay of motor memories. As long as the error was removed, but participants did not think that the context had changed, full motor memory was retained, immune from decay. However, this view was recently challenged^43^, where they showed that the maintenance of the motor memories in the previous study^42^, termed the context-dependent decay hypothesis, was due to the correlated noise between subsequent channel trials and that motor memories decay irrespective of a specific change in context. Our results contrast in that we propose that different contexts, (produced through different plans rather than simply the detection of a change in the dynamics) activate distinct motor memories, each of which would only decay in the absence of error during their activation. Simply detecting a change in the dynamics may not be sufficient to switch contexts.

The specific conditions needed to ensure the protection of motor memories are still a matter of ongoing research. It has been suggested that the presence of reinforcement or reward^44^ is a critical signal that affords protection to the motor memories. Other researchers have argued that motor learning should be addressed as a Bayesian inference problem^45, 46^. Within this framework, they derive a probabilistic source estimation model that separates contributions from the body and world using the discrepancies between the observed and predicted movements. They suggest that the sensorimotor control system stores and retrieves movement parameters based on their possible long-term relevance, and that protection of motor memories should depend on the likelihood that these memories are relevant for future actions.

In summary, we demonstrated that lead-in variability affects the speed of motor learning, but that this effect depends on the sensory modality with only variability in active lead-in movements reducing the learning rate. This further supports the previous observation that different sensory modalities have distinct generalization characteristics that can dramatically influence the rate of motor adaptation, which is a critical factor for both skill learning and rehabilitation.

## Methods

### Single rate state-space model

To implement a model exhibiting angular selectivity, we use a set of basis functions, one per state, uniformly spread around the unit circle. Here we used a total of 36 bases yielding peak responses separated by 10°. We note that using more or fewer basis functions does not affect the results of the simulation. To represent these multiple overlapping states we use a vector Z(t), where t represents trial index and each element in the vector represents a separate state. We also define a context vector C(θ_lead-in_) to weight the state contributions as a function of lead-in angle θ_lead-in_. Each element c_i_ in the context vector is given by:

1 $${c}_{i}=A\,\exp (k\,\cos ({\theta }_{lead-in}-\,{\theta }_{i}))$$

here the subscript i represents the base element index, the term (θ_lead-in_ − θ_i_) represents the deviation angle of the lead-in movement direction from the center of the receptive field of the given base, A is a scaling factor such that the receptive field function peaks at unity, and k is the von Mises tuning width parameter which relates to the Gaussian distribution variance by the expression k = 1/σ^2^.

The overall net output x(t) at trial t is given by the scalar product of the state vector and the normalized context vector. Note that the normalization term is necessary so that the value of the net output cannot exceed unity. This ensures that the net output is matched to the perturbation term (see below) and that changes in tuning width do not affect the final asymptotic level of learning. The net output x(t) is given by:

2 $$x(t)=Z{(t)}^{T}\cdot \frac{C({\theta }_{lead-in})}{\sum C({\theta }_{lead-in})}$$

The presence or absence of a novel dynamic perturbation (curl field) at trial t is represented by f(t). In the null field condition f(t) = 0 while in the curl field f(t) = 1. The error e(t) at trial t is the difference between force field f(t) and the net output of the model x(t):

3 $$e(t)=f(t)-x(t)$$

The update equation for the state vector Z(t) consists of the sum of two terms. The first term represents a decaying memory of the previous state vector implemented using an element-wise product of a contextually-dependent retention vector term α(θ_lead-in_) and the current state. The second term is a update contribution to the current state arising from the error e(t) experienced on that trial weighted by a learning rate β and the context vector:

4 $$Z(t+1)=\alpha ({\theta }_{lead-in})\cdot Z(t)+\beta \cdot e(t)\cdot C({\theta }_{lead-in})$$

The retention vector α(θ_lead-in_) needs to be contextually dependent so that states (i.e. memories) that are not visited do not decay. That is, a memory that is not disturbed remains intact. This ensures that asymptotic levels of compensation during field exposure are independent of the level of lead-in diversity. Here we use a simple expression to compute α(θ_lead-in_). It sets retention to unity when there is zero contextual activation of a state (i.e. it has perfect memory when the state is not involved in the trial). As the contextual activation for a state approaches unity, the term α(θ_lead-in_) tends to a lower minimum value α_min_ (i.e. when the state is fully involved, a standard retention rate is used). Each element in α _i_ the retention vector is thus given by:

5 $${\alpha }_{i}=1+({\alpha }_{min}-1){c}_{i}$$

where c_i_ is the element i in the context vector. Since the pattern of activation of the context vector is used to compute the net compensation and update the state, it plays a key role in how fast the model learns.

## Experimental Design

Seventy-two participants (26 male, 46 female; age = 23.1 ± 5.8 years, mean ± sd) were randomly allocated to six experimental groups. All participants were right handed according on the Edinburgh handedness questionnaire^47^, and were naïve to the aims of the study. All participants provided written informed consent to the protocol, which had been approved by the University of Plymouth Faculty of Science and Technology Human Ethics Committee. The methods were carried out in accordance with the approved guidelines.

### Apparatus

Experiments were performed using a vBOT planar robotic manipulandum and its associated virtual reality system^48^. Handle position is measured using optical encoders sampled at 1000 Hz, and motors operating under torque control allow the application of end-point forces. A force transducer (Nano 25; ATI), mounted under the handle, measures the applied forces, and its output signals were low-pass filtered at 500 Hz using analogue 4th pole Bessel filters prior to digitization. To reduce body movement participants were seated in a sturdy chair in front of the apparatus and firmly strapped against the backrest with a four-point seatbelt. During an experiment, participants held the robot handle in their right hand while their right forearm was supported by an air sled, constraining arm movement to the horizontal plane. Participants could not see their hand directly. Instead veridical visual feedback was used to overlay images of the starting location, via point, final target, (all 1.25 cm radius disks) and a hand cursor (0.5 cm radius red disk) using the virtual reality system. This ensured that the visual cursor appeared to the participant in the same plane and at the same location as their hand. Data was collected at 1000 Hz and logged to disk for offline analysis using Matlab (Matlab, The MathWorks Inc., Natick, MA, USA).

### Protocol

The experimental paradigm was designed to examine how changes in lead-in movement diversity and modality affect the rate of learning of a single force field. All experiments, except the control condition, consisted of two part movements. The first part constituted a contextual movement (lead-in movement to a central position) and the second part an adaptation movement (movement to the final target). During the adaptation movement, participants experienced a single force field, the direction of which was fixed for the duration of a given experiment, but randomized across subjects.

In separate experiments, we examined active and visual lead-in movements. For both of these lead-in modalities, the effect of changing lead-in movement diversity was examined using different ranges of starting locations (Fig. 2). These lead-in movements began 10 cm from the central position on the circumference of a semicircle. The range of the lead-in conditions varied between 180° and 0°. The direction of the force field and lead-in movements was counter-balanced across participants. For comparisons, a control condition was also examined, in which there were no lead-in movements. Participants were randomly allocated to these six different experimental groups.

### Force Fields

In the adaptation movement, participants performed reaching movements either in a null field condition, a velocity-dependent curl force field^49^, or a mechanical channel^39^. The curl force field was implemented as:

6 $$[\begin{array}{c}{F}_{x}\\ {F}_{y}\end{array}]=k[\begin{array}{cc}0 & -1\\ 1 & 0\end{array}]\,[\begin{array}{c}\dot{x}\\ \dot{y}\end{array}]$$

where the field constant k was set to a value of ±16 N m^−1^ s (the sign determines the direction (CW or CCW) of the force-field). Each participant only experienced a single force field direction. Mechanical channel trials were implemented using a spring constant of 6,000 N/m and a damping constant of 30 Nm^−1^ s perpendicular to the direction of motion throughout the movement between the central location and the final target. Channel trials were always associated with a lead-in movement from either +45° or −45°, with the direction counterbalanced across participants. The direction of contextual movements and curl field direction (CW/CCW) was counterbalanced, with half the participants in a given experimental group randomly assigned a CW curl field, and the other half a CCW curl field.

#### *Experiment 1. Active lead-in movements* (*n* = *36*, *3 groups*)

In this experiment, the contextual lead-in movement was an active movement performed by the participant with a visible cursor. On the two-part movements, the start location for the lead-in movement, the central location and final target were initially displayed. The vBOT then moved the participant’s hand to the lead-in start location following a minimum jerk trajectory. Once the handle was stationary within the location (speed < 0.1 cm/s for 500 ms), a beep was presented indicating the start of the trial. This active lead-in movement to the central position was always performed in the null force field. Once the cursor reached the central location, participants were encouraged to pause briefly (speed < 5 cm/s for 50 ms) before making the second movement to the final target location, otherwise a warning was provided. If the second movement (adaptation movement) duration was between 150 ms and 300 ms a “correct speed” message was displayed; otherwise an appropriate “too fast” or “too slow” warning was shown. Force fields and channel trials were only ever presented during this second movement.

Three groups of participants were used to examine the effect of lead-in diversity (consistent, medium variability and high variability). In the consistent lead-in group (Fig. 2a), the lead-in movement was always made from the same start position (counterbalanced at +45° or −45° across participants). In the medium lead-in variability group (Fig. 2b), the lead-in movement on each trial was pseudo-randomly selected from a set of 9 equally spaced starting locations between −90° to 0° or 0° to 90° (ranges counterbalanced across participants). In the high variability lead-in group (Fig. 2c), the lead-in movement on each trial was pseudo-randomly selected from a set of 9 equally spaced starting locations between −90° to +90°.

#### *Experiment 2. Visual lead-in movements* (*n* = *24*, *2 groups*)

In this experiment the contextual lead-in movement was a purely visual movement of the cursor, which followed a minimum jerk trajectory of duration 640 ms from the start to the central location, during which time the participant’s hand remained stationary at the central location. Immediately after the cursor reached the central location the participant made a reaching adaptation movement from the central location to the final target. The dwell time of the cursor within the central location was required to be 50 ms (speed < 5 cm/s for 50 ms). Two ranges of lead-in diversity were used in this experimental condition, namely a consistent lead-in (Fig. 2d) and a high variability lead-in (Fig. 2e). All other conditions are identical to the first experiment.

#### *Experiment 3. Control with no lead-in movement* (*n* = *12*, *1 group*)

In this control experiment, the participants did not experience a contextual lead-in movement (lead-in start location also not shown) and only made a single reaching adaptation movement from the central location to the final target (Fig. 2f) after the initial beep was presented. All other conditions for the adaptation movement were identical to the other two experiments.

### Trial Order

All experiments were organized in 75 blocks of 10 trials. A block consisted of pseudo-randomly ordered trials comprising nine null/field trials and a single channel trial. Each experiment began with a pre-exposure phase consisting of 300 trials in which no force field was applied (null field). This was followed by an exposure phase of 400 trials in which the adaptation movement occurred in the curl field). Finally there was a post-exposure phase consisting of 50 trials (null field). Participants were provided with short rest breaks approximately every 200 trials (195–205 trials), but could take a break at any time.

As we specifically wished to examine the learning rate, we took several precautions with the experimental design. First, a long period of pre-exposure (300 trials) was provided such that participants could reliably perform the task. During this pre-exposure phase, any critical errors resulted in aborting the trial. These critical errors included moving towards the final target before the cursor reached the central location (visual condition) or waiting too long at the central position (dwell times >300 ms). Controlling the dwell time was important as large dwell times diminish the contextual effect of lead-in movements^13^. However, during the exposure phase of the experiments, only warnings were generated in these conditions and trials were not aborted. This ensured that the learning rate was not affected by any aborted trials.

## Analysis

The experimental data was analyzed offline using Matlab. To examine learning, kinematic error on the adaptation movements and force compensation on the channel trials were used.

### Kinematic error

For each non-channel trial, the kinematic error was calculated on the adaptation portion of the movement as the maximum perpendicular error (MPE). The MPE is the maximum deviation of the hand path to the straight line joining the movement starting location to the centre of the target. For each participant, a 10 trial running mean was calculated. The MPE sign was flipped appropriately so that results from CW and CCW field trials could be appropriately combined. The mean and standard error (SE) of MPE was then computed across all participants.

### Force compensation

On each channel trial, we measured the force compensation to look for evidence of predictive feed-forward adaptation, as opposed to relying on a reduction in kinematic error during force field learning which can also arise from muscle co-contraction^50–52^. For channel trials, the force produced by participants perpendicularly into the wall of the simulated channel was regressed with the velocity of movement along the channel during the same period multiplied by the field strength. This yielded an estimate of the level of force compensation present at the given channel trial^53^. For plotting purposes, the force compensation data was averaged. For each participant, the running mean of 10 channel trials was calculated. The mean and standard error (SE) of compensation was then computed across all participants.

### Estimation of learning rate

The learning rate was estimated for the 6 different experimental conditions by fitting an exponential function to individual participant force compensation data using non-linear optimization (Matlab function nlinfit). The compensation during field exposure is modelled using the expression:

7 $$compensation=A[1-\exp (-tB)]$$

where t is the trial index, A is a constant representing the asymptotic level of compensation reached and B = 1/tau, where tau is the learning rate over trials. This procedure obtained the best fitting trial constant and asymptote, which represents that final level of force compensation for each participant. The mean and SE of these parameters across participants was then calculated for each experimental condition.

### Statistics

Statistical analysis was performed in SPSS 21.0 using the generalized linear model to perform an ANOVA. If main effects were significant, then post-hoc comparisons were performed using Tukey’s HSD test. Significance was considered at the p < 0.05 level.

Since learning can also potentially be affected by movement kinematics, especially dwell time^13^, we performed an analysis of movement kinematics from the central location to the final target during the pre-exposure trials. We measured the dwell time, the lateral deviation of the adaptation movement, and the peak speed. Differences were examined using an ANOVA. Comparisons were made across all six conditions in the three experiments with the exception of dwell time for which the control experiment was excluded).
